# Development of a Cocreated Perioperative Joint Replacement Digital Care Pathway to Improve Surgical Outcomes Following Joint Replacement: Protocol for a Mixed Methods Study

**DOI:** 10.2196/85701

**Published:** 2025-12-04

**Authors:** Adam I Semciw, Rebecca Jessup, Rachel Duckham, Swapna Gokhale, Hayley Gray, Emily C Bell, Stephen M Quick, Juliette Gentle, Uyen Phan, Katharine See, Rebecca Galea-O’Neill, James Boyd, Hazel Heng, Tracey Webster, Matthew G King

**Affiliations:** 1 La Trobe University Bundoora Australia; 2 Northern Health Epping Australia; 3 The Royal Melbourne Hospital Melbourne Australia; 4 The University of Melbourne Melbourne Australia; 5 Deakin University Melbourne Australia

**Keywords:** arthroplasty, rehabilitation, co-design, digital health, patient education

## Abstract

**Background:**

Recently, perioperative care has gained attention for its ability to improve outcomes, reduce costs, and enhance patient satisfaction, especially when multidisciplinary support is involved. Despite these benefits, patient compliance remains low due to limited engagement in program design and practical barriers such as transportation, particularly for older adults. Co-designed digital health solutions offer a promising, scalable approach to delivering personalized, accessible perioperative care, with emerging evidence supporting their feasibility and effectiveness in patients who undergo joint replacement.

**Objective:**

The objectives of this study protocol are to outline the methods to address three study aims: (1) understand gaps and unmet needs, including knowledge, perceptions, barriers, and acceptability, during the perioperative patient journey of hip and knee arthroplasty; (2) co-create a novel patient-centric digital care pathway (DCP) that provides education and systematically captures patient-reported outcomes; and (3) evaluate the feasibility of implementation, appropriateness, and acceptability of the pathway when tested in patients undergoing nontraumatic hip and knee joint arthroplasty.

**Methods:**

This mixed methods co-design and implementation study will be conducted across 3 phases informed by the generative co-design framework for health care interventions. In phase 1 (predesign), patient interviews and journey mapping will be used to identify perioperative care gaps to be addressed in the DCP. In phase 2 (co-design), care gaps will be collaboratively framed, and iterative prototyping of the DCP will be conducted with consumer feedback and pilot testing. In phase 3 (evaluation), the feasibility of the DCP will be assessed using a previously reported framework. Inclusion criteria will vary across phases, focusing on people with lived experience or undergoing hip or knee joint replacement and relevant clinical or administrative staff.

**Results:**

The study was funded in September 2024, with phase 1 commencing in July 2025 and phase 2 in October 2025. Phase 3 is projected to commence before the end of 2025 and conclude 6 months later. As of October 2025, 13 people had been recruited, interviews were completed for phase 1, and recruitment for phase 2 had commenced.

**Conclusions:**

This protocol enhances methodological transparency by detailing the co-design approach, strengthening the evidence base, and supporting the development of a credible, transferable DCP. It addresses the lack of patient-centric perioperative programs in joint replacement care by incorporating individual needs and preferences. The digital format helps overcome access barriers, such as transport limitations, enabling patients to engage with the pathway anywhere. Additionally, patient-reported outcome measures collected through the DCP will improve understanding of recovery trajectories following hip and knee replacements.

**International Registered Report Identifier (IRRID):**

DERR1-10.2196/85701

## Introduction

Surgery plays a major role in health care systems worldwide, with an estimated 310 million surgeries performed globally each year [1,2]. However, surgery is associated with significant risks and the burden of complications [1,2]. Despite advances in surgical technology, anesthesia, and perioperative care, postoperative complications remain common. For orthopedic surgery, 20% to 40% of people experience postoperative complications manifesting in reduced functional and psychological capacity, such as fatigue, sleep disturbance, and decreased quality of life [2-4]. These complications lead to poor health outcomes, prolonged hospital stays, and high readmission rates [5-7].

Over the past decade, perioperative care strategies have received greater attention as a mechanism to improve postoperative outcomes [8]. High-quality perioperative care reduces health care costs, length of stay, and postoperative complications, while simultaneously improving patient satisfaction [4,8-10]. Patients generally report high satisfaction with these interventions, particularly when multidisciplinary support is provided [11,12]. However, despite these reported benefits, patient compliance remains low (<50%) [11]. This is often attributed to the absence of engagement with end users in their design and development, leading to a lack of patient-centric programs that consider patients’ individual needs and preferences [11,13]. This is further inhibited by practical barriers, such as travel and transport difficulties, particularly among older adults [14]. Patients want early, comprehensive information and support for preoperative preparation [2,15], often resorting to self-directed efforts or information from their general practitioner [2], highlighting a gap in readily accessible, patient-centered pathways for these patients.

Co-designed digital health solutions have been proposed as practical and scalable methods to address barriers to accessing and completing perioperative care. Digital health, using information and communication technologies, has the potential to deliver personalized, accessible, and patient-centered perioperative care that patients can engage with at their own pace, regardless of location [16,17]. Emerging evidence highlights that digital prehabilitation interventions are acceptable and feasible for people preparing for hip and knee arthroplasty [2,17-19], with telehealth rehabilitation demonstrating comparable clinical and patient-reported outcomes to in-person physical therapy [20,21]. However, there remains limited research examining the codevelopment of digital health resources and their successful implementation within public health services [13,19]. A codeveloped digital care pathway (DCP) may augment patient monitoring and provide effective perioperative clinical care for people undergoing hip and knee arthroplasty. A DCP is an application-based digital adjunct used to facilitate patient care [22]. This enables the delivery of accurate and relevant education and self-management resources while also facilitating the routine collection of patient-reported outcomes, thereby facilitating the early identification of patients at risk of poor long-term outcomes [22,23]. Therefore, the aims of this study are as follows:

1. Understand gaps and unmet needs, including knowledge, perceptions, barriers, and acceptability, during the perioperative patient journey of hip and knee arthroplasty.
2. Co-create a novel patient-centric DCP that provides education and systematically captures patient-reported outcomes.
3. Evaluate the feasibility of implementation, appropriateness, and acceptability of the pathway when tested among patients undergoing nontraumatic hip and knee arthroplasty.

## Methods

### Study Design

This mixed methods co-design and implementation study will be reported in accordance with the STROBE (Strengthening the Reporting of Observational Studies in Epidemiology) statement [24]. To address the 3 distinct aims, the study will be conducted across 3 phases informed by the generative co-design framework for health care interventions [25], as outlined in Textbox 1 [26-29]. A learning community will be established, comprising patients, health care staff, administrators, and researchers, to inform and guide each stage of the co-design process.

**Phases of co-design used to develop the digital care pathway.**

**Phase 1: Predesign (understand)**

- Contextual inquiry: patient journey mapping [26-28] to describe the perioperative pathway and identify service gaps, and interviews with consumers. Reporting informed by prompts from Davies et al [26].
- Preparation: establishment of a learning community involving patients, staff, administrators, and researchers.

**Phase 2: Co-design**

- Framing issue: identification of care gaps and collaborative development of potential solutions.
- Generative design: iterative prototyping of digital care pathway features with feedback from the learning community.
- Sharing ideas: pilot-testing of the refined prototype with a sample of patients.

**Phase 3: Postdesign (evaluate)**

- Data analysis: assessment of feasibility using the framework for feasibility studies by Bowen et al [29].
- Requirements translation: conversion of co-design outputs into workflows and technical specifications, aligned with perioperative processes.

### Ethical Considerations

This study has been approved by the St Vincent Hospital Human Research Ethics Committee (2024/PID00354 LRR 288/24) under the National Health and Medical Research Council of Australia National Mutual Acceptance Scheme. The study has also received research governance approval from La Trobe University (LRR 288/24), with Northern Health research governance to be sought before any direct recruitment of Northern Health staff or patients. Participants will provide consent as follows: (1) written informed consent for patient and staff interviews, and (2) implied consent for the use of data generated through the completion of the DCP. Participants’ privacy and confidentiality will be maintained in accordance with ethical approval conditions and local laws. People with lived experience of joint arthroplasty that participate in the interviews in phase 1 or phase 3 will receive an Aus $20 (US $13.07) gift card following the interview. Individuals who participate in the DCP, as well as organizational and clinical staff, will not receive compensation for their participation.

### Setting

The study will be conducted at Northern Health, Victoria, Australia [30]. Northern Health is the major provider of acute, subacute, and specialist services in Melbourne’s rapidly growing outer north corridor. The health service caters to a diverse population of patients who speak more than 100 languages. The catchment has lower levels of income, educational attainment, and health literacy and higher rates of unemployment than the Victorian state averages [31]. Northern Health has pioneered digital health integration through initiatives such as the Victorian Virtual Emergency Department [32] and DCPs [22].

### Current Joint Arthroplasty Touchpoints With Opportunities for a DCP

**Figure 1:**
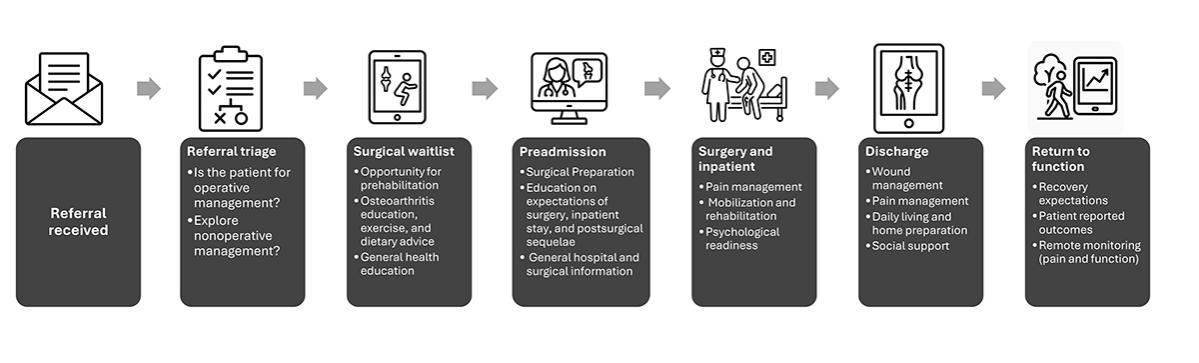
High-level overview of key touchpoints with elective joint replacements in the public health service and a potential perioperative digital care pathway.

The key touchpoints associated with elective arthroplasty in the public health service are illustrated in Figure 1. Each touchpoint provides an opportunity to reinforce education provided by clinicians through electronic or digital resources, codeveloped with patients. For example, when patients are deemed suitable for surgery, there can be long wait periods from the preadmission clinic to the surgery date. This provides an opportunity to deliver evidence-based education and structured prehabilitation, aimed at optimizing physical function, managing comorbidities, supporting psychological readiness, and promoting healthy lifestyle behaviors. At the preadmission clinic, patients could receive additional resources to support their preparation for surgery. These resources might include information on pain management strategies, expectations regarding acute recovery, guidance on postoperative mobilization, and discharge destination. Other potential resources, highlighted in a recent systematic review, that have been used in prehabilitation include, but are not limited to, general hospital and surgical information, daily living and home preparation, and psychosocial and supportive aspects (eg, to reduce fear and anxiety around surgery) [10]. The integration of patient-reported outcomes and remote patient monitoring could enable patients and clinicians to follow the trajectory of recovery with benchmarked standards [33].

### Participants

Formal participant inclusion and exclusion criteria for phases 1 and 2 of the study are outlined in Textbox 2. For phase 2, people with lived experience of arthroplasty from phase one, as well as Northern Health staff working in perioperative hip or knee arthroplasty surgical care, will be invited to participate in co-design workshops and provide feedback on the development of the DCP consistent with the co-design steps of the generative co-design framework for health care innovation [25].

**Overview of phase 1 (understand) and phase 3 (evaluate) inclusion and exclusion criteria.**

Phase 1

- People with lived experience
  Inclusion criteria: individuals aged 18 y or older who have undergone hip or knee arthroplasty surgery in the last 12 mo
  Exclusion criteria: unable to understand spoken or written English
- Organizational and clinical staff
  Inclusion criteria: not applicable
  Exclusion criteria: not applicable

Phase 3

- People with lived experience
  Inclusion criteria: any patient undergoing hip or knee arthroplasty surgery at Northern Health in the first 6 mo of implementing the digital care program into standard care
  Exclusion criteria: unable to understand spoken or written English
- Organizational and clinical staff
  Inclusion criteria: any primary or secondary contact clinician working in perioperative hip or knee arthroplasty surgical care at Northern Health, or any administrator working in perioperative hip or knee arthroplasty surgical pathway at Northern Health
  Exclusion criteria: unable to understand spoken or written English

### Procedures

#### Phase 1: Understand

##### Step 1: Contextual Inquiry

People with lived experience of a total hip or knee arthroplasty will be recruited via widespread print and social media advertising. Semistructured interviews (Multimedia Appendix 1) will be conducted to encapsulate their perioperative journey, with a focus on barriers, enablers, gaps, and unmet needs across common care touchpoints. The interviews are expected to capture the physical (functional) and emotional (experiential) dimensions of care, providing insights into common patient experiences along their care pathway. The interviews aim to elicit the behaviors, feelings, motivations, and attitudes of people with lived experience across their entire episode of care [26].

This interview guide has been developed using the capability, opportunity, motivation-behavior (COM-B) model of behavior change [34]. COM-B is a theoretical framework that proposes that any behavior (B) results from the interaction of 3 components: capability (physical and psychological), opportunity (social and environmental factors), and motivation (reflective and automatic processes) [34]. Drawing on this framework ensures that the interview questions address a broad range of influences on patient behavior, including functional abilities, social context, and motivational drivers. In this study, COM-B will be used solely to inform the design of interview questions, ensuring comprehensive coverage of factors that may shape patient experiences across their perioperative journey.

**Figure 2:**
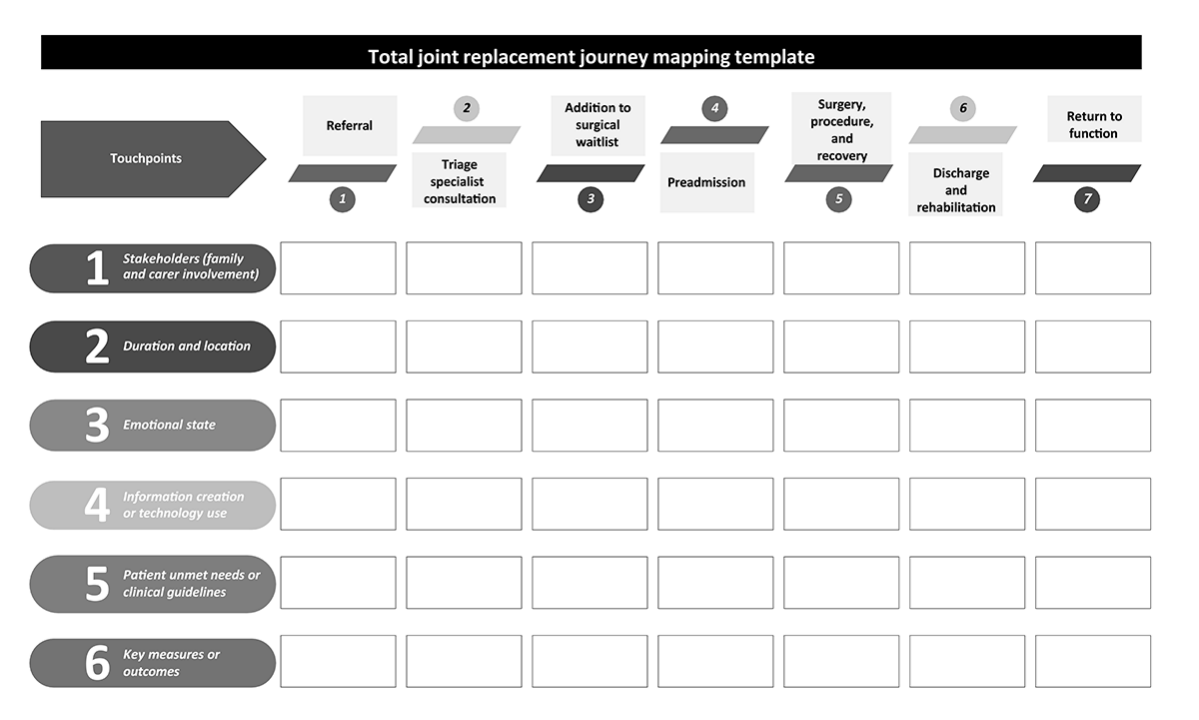
Overview of the proposed journey mapping touchpoints.

Following data collection, qualitative data from the interviews will be imported into NVivo (QSR International) and analyzed using the framework method (transcription, familiarization, coding, developing an analytic framework, indexing, data charting, mapping, and interpreting) [35]. We will use a combined inductive and deductive coding approach, guided by the 6-step method developed by Terry et al [36]. In the first round, 2 researchers will familiarize themselves with the data by reading through all transcripts (step 1). They will then independently inductively code all transcripts (step 2). Following this, the researchers will meet to compare and collapse all similar codes (step 3) and will refine them until there is a final, agreed-upon set of codes (step 4). In the second round of coding, the agreed-upon codes will be applied to the entire dataset independently by the 2 researchers (step 5). In the final round, themes will be visually concept-mapped across critical touchpoints, barriers, and enablers to develop an initial journey map of the patient experience (Figure 2).

##### Step 2: Preparation

A learning community will be established, comprising key stakeholders, including people with lived experience of arthroplasty, system-level representatives (eg, health service managers), clinicians (eg, physiotherapists, surgeons, and nurses), and administrators and researchers. Accessibility requirements will be established during this step, and we will purposively include consumers with diverse literacy levels, cognitive needs, physical limitations, and cultural backgrounds. As part of the co-design process, we will identify barriers to digital engagement, preferred modes of interaction, and required functional supports. Members will be oriented to the project, provided with relevant resources, and prepared for participation in subsequent phases of the co-design process. Recruitment will draw on participants from step 1, as well as word-of-mouth, professional networks, and organizational invitations. These stakeholders will be embedded in each decision-making stage and contribute to the co-creation process, as appropriate.

#### Phase 2: Co-create

##### Step 3: Framing the Issue

Insights from step 1 will be synthesized to identify gaps in perioperative care and potential areas for service improvement. Preliminary models, pathways, and resources will be developed based on these findings and used to frame key issues for discussion with the learning community. This process will ensure that the problems and priorities reflect both the lived experience and system perspectives.

##### Step 4: Generative Co-Design

Draft pathways and resources will be presented to the learning community during a series of workshops or interviews, conducted either face-to-face or by videoconference. Participants will collaboratively critique and refine these prototypes, providing feedback on feasibility, usability, and acceptability. Iterative adjustments will be made following each session, resulting in progressively refined pathway models and supporting resources. During the DCP development, we will ensure alignment of content and design with web accessibility standards and health literacy design principles. This will include ensuring that the digital interfaces use plain language (tested using the Flesch Kincaid readability test and will aim for a grade level of 5), closed captions for multimedia (available in multiple languages), and easy-to-see and hear text content using preferred fonts for readability, accessible colors, and sufficient contrast.

##### Step 5: Sharing Ideas

The draft DCP prototype will be implemented on a small scale in patients undergoing total knee or hip arthroplasty at Northern Health. This stage will provide an opportunity to share the model with patients and staff, observe how it operates in practice, and capture early feedback on its use within standard care. All patients triaged for arthroplasty during the first 6 months of implementation will be invited to participate in the pathway. Insights from this process will inform the refinement of the pathway and identify practical considerations to be explored in the evaluation stage.

#### Phase 3: Postdesign (Evaluate)

##### Step 6: Data Analysis

###### Overview

Data generated during the pilot implementation (step 5) will be analyzed to assess the feasibility of introducing the DCP into routine perioperative care. A mixed method data analysis will be used to explore the acceptability, appropriateness, barriers, and enablers to implementation.

Feasibility will be assessed using a mixed methods approach informed by the framework developed by Bowen et al [29]. This will include the domains of demand, acceptability, implementation and practicality, and pilot effectiveness of the DCP. Notably, the assessment of these domains during the initial 6-month period is to understand the DCP’s initial implementation. The intention is developmental, supporting iterative refinement, and identifying additional resource needs and allocations, rather than determining the DCP’s overall success.

###### Demand

Demand will be based on uptake of the DCP from those invited to participate (all individuals undergoing a hip or knee arthroplasty in the first 6 months of implementation). Demand outcomes will include application downloads, completion of the patient-reported outcomes, and resource views.

###### Acceptability

We will assess dimensions related to the Theoretical Framework of Acceptability by Sekhon et al [37], including confidence in recommending to family and friends, the level of burden associated with the DCP, and whether the desired outcome was achieved. These will be assessed using a survey administered by Research Electronic Data Capture at the preadmission clinic and 12 weeks after discharge.

###### Implementation and Practicality

We will assess implementation and practicality via semistructured interviews (Multimedia Appendix 2) with staff and individuals with lived experience to explore their perspectives to identify factors influencing implementation, resource requirements, and timeliness of delivery.

###### Pilot Effectiveness

Patient-reported outcomes will assess pain, function, and quality of life over time using the short form hip or knee osteoarthritis outcome score [38], with additional measures to be informed by patients as part of phase 2. This will be assessed at the following time points (before surgery, discharge, 2 weeks after surgery, 6 weeks after surgery, 12 weeks after surgery, and 6 months after surgery).

###### Demographic Analysis

Participant demographics and baseline characteristics will be reported using descriptive statistics, as appropriate, based on normality.

###### Demand and Acceptability Analysis

Outcomes will be reported as counts, frequencies, and proportions or by parametric or nonparametric descriptive statistics as appropriate based on the individual outcome and normality.

###### Implementation and Practicality Analysis

Semistructured interview recordings will be transcribed and provided to the participant within 14 days for the participant to confirm that the data reflects their perspective. The transcript will be deemed accurate if no feedback is received within 14 days. Data will be analyzed using reflexive thematic analysis [39] to identify themes, with coding conducted by 2 independent researchers and compared to identify present themes [39].

###### Limited Efficacy and Effectiveness Analysis

Patient-reported outcome measure data will be analyzed in accordance with previous DCP evaluations [22]. Recovery trajectories of patient-reported outcomes will be summarized using linear mixed-effects models to account for missing data. Data will be presented as linear or nonlinear trajectories based on the best model fit (as assessed by the Akaike information criterion) [40]. Full information maximum likelihood will be used to account for missing data. All statistical analyses will be conducted using R statistical software (R Foundation for Statistical Computing) with an alpha value of .05.

###### Sample Size: Patient and Staff Interviews

Interviews will continue until data saturation is reached. Saturation will be predefined as 3 consecutive interviews where no new theoretical categories are identified past an initial minimum sample of 12 [41,42].

###### Sample Size: DCP Pilot Implementation

No priori sample size was defined for DCP completion, consistent with feasibility study objectives.

##### Step 7: Requirements Translation

Findings from the pilot evaluation will be synthesized into a finalized model of the DCP. This model will integrate the requirements articulated by co-design participants with the practical considerations identified during the evaluation (eg, feasibility, acceptability, workflow fit, and resourcing). In line with guidance on feasibility and implementation outcomes [29,43], the evaluation will determine which components are core and must be preserved, and which can be adapted for local contexts. Processes, workflows, and supporting materials will be refined to ensure that the DCP is ready for translation into routine practice.

## Results

The study was funded in September 2024, with phase 1 commencing in July 2025 and phase 2 in October 2025. Phase 3 is projected to commence before the end of 2025 and conclude after 6 months of recruitment. As of October 2025, 13 participants were recruited. Interviews were completed in phase 1 and recruitment for phase 2 commenced.

## Discussion

### Anticipated Findings

Developing a protocol that explicitly outlines the co-design methods proposed for the DCP is important, given the inconsistent reporting evident in the existing literature. The umbrella review by Kilfoy et al [13] highlighted that while co-design is widely promoted as a strategy to enhance the effectiveness and sustainability of digital health interventions, its application is inconsistently reported, with details of participant involvement and methodological rigor often lacking. The absence of clear reporting impedes the evaluation, reproducibility, and progression of existing work, restricting the incorporation of equity and stakeholder perspectives in digital innovation. By specifying our co-design approach, this protocol contributes to methodological transparency, strengthens the evidence base, and ensures that the resulting pathway is both credible and transferable to other contexts and settings. Therefore, we present our study protocol for this mixed methods co-design and implementation study that aims to understand the lived experience of people with knee and hip arthroplasty, co-create a DCP and evaluate its feasibility and appropriateness of implementation.

This study will inform the development and ongoing iterative improvements of a DCP for people undergoing arthroplasty, underpinned by the engagement with end users. This engagement aims to address the lack of patient-centric programs in perioperative joint replacement care and allow the health services to better consider patients’ individual needs and preferences [11,13]. The digital nature of the care pathway aims to address access barriers associated with a lack of transportation [14] and allows patients to engage with their ongoing care at any time, regardless of their location [16,17].

The patient-reported outcome measures collected through this DCP will provide a greater understanding of symptom recovery trajectories of people undergoing hip and knee arthroplasty. Our current understanding is limited to isolated studies [33], which may not reflect the current patient outcomes. We hope to supplement this work by capturing rich, real-time data, allowing patient recovery to be mapped and compared with benchmarked trajectories over time. The data obtained will also inform patient education regarding realistic recovery expectations and timelines, and underpin the development of further resources to support this. Although this study aims to evaluate the DCP over the first 6 months of its implementation, the objective is to integrate the pathway into ongoing standard-of-care practices. As a result, the DCP will undergo continual refinement on an iterative basis to adapt to the changing landscape of the health care system [22].

### Conclusions

This mixed methods co-design implementation study is consumer-driven, with patients and carers actively involved from problem identification to solution design and evaluation. This approach ensures that the development of a DCP for joint replacement is grounded in lived experience, addressing real gaps in care and unmet needs. By embedding co-design principles and drawing on established frameworks, this study privileges consumer voices, promotes collaboration between patients and clinicians, and increases the likelihood that the resulting pathway will be acceptable, relevant, and sustainable in practice.

## Data Availability

Data sharing is not applicable to this article as no data sets were generated or analyzed during this study.

## Appendix

Multimedia Appendix 1 Patient interview guide for phase 1.

Multimedia Appendix 2 Patient interview guide for phase 3.

## Abbreviations

COM-B: capability, opportunity, motivation-behavior
DCP: digital care pathway
STROBE: Strengthening the Reporting of Observational Studies in Epidemiology
