# Supplementary material for: Development of a Cocreated Perioperative Joint Replacement Digital Care Pathway to Improve Surgical Outcomes Following Joint Replacement: Protocol for a Mixed Methods Study
Source: JMIR Res Protoc. 2025 Dec 4;14:e85701. doi: 10.2196/85701 (PMC12715470; doi:10.2196/85701)
Supplement: Multimedia Appendix 1 [file resprot_v14i1e85701_app1.docx]

# Patient Interview Guide – Phase 1

The aim of this interview is to explore patients’ perspectives of their perioperative care for orthopaedic surgeries. We are interested in understanding the knowledge, attitudes, facilitators and barriers to engaging with the perioperative pathway.

| Age |  |
| --- | --- |
| Gender and Sex | Male  Female  Other |
| Surgery type |  |
| Aboriginal or TSI | Yes  No  Prefer not to say |
| Is English your primary language spoken at home? | Yes  No |
| If no, what is your primary language spoken at home? |  |
| Employment Status | Full time  Part time  Casual  Unemployed  Retired |
| Partnered/non-partnered/Living arrangement |  |
| What is your highest education level? | Year 12 or equivalent  Diploma  Undergraduate  Postgraduate  Other |
| If other, what is your highest education level? |  |
| How long have you had your pain for? | Under one year  1 – <3 years  3 - <5 years  5 years or more |

Interview Guide*

*This is a guide only and questions may change based on the direction the participant wants to take the interview. The aim is to gain a rich insight into the patient’s true experience of perioperative management.

| COM-B Framework  Question Area | Suggested Questions | Prompts |
| --- | --- | --- |
| Introductory question |  |  |
| Capability | Could you tell me about what stands out for you or what you remember most clearly about waiting to have surgery? | What life impact did waiting for surgery have for you? How long did you have to wait from initial referral to seeing a surgeon, from seeing the surgeon to surgery? |
|  | Did you feel physically prepared for the surgery and recovery process? |  |
|  | Were there any physical challenges that made it difficult to follow pre- operative instructions? |  |
|  | How well did you understand the information provided about your surgery and recovery? |  |
|  | Did you feel confident in managing your care before and after the surgery? |  |
|  | Were you able to remember and follow the instructions given by your healthcare team? |  |
| Opportunity | Did you feel supported by family, friends, or caregivers during your surgical journey? |  |
|  | How would you describe your interactions with healthcare staff? | Did you feel staff communicated well? Did you feel cared for? |
|  | Did cultural or language differences impact your understanding or comfort during the process? |  |
|  | Was the hospital environment and its facilities accommodating to your needs? |  |
|  | Were there any logistical challenges, such as transportation or accessibility, that affected your experience? |  |
|  | Did you have access to the tools or equipment you needed for recovery? | For example: mobility aids, pain management supplies? |
| Motivation | What motivated you to undergo this surgery? |  |
|  | Did you believe that the surgery and recovery plan would help you achieve your health goals? |  |
|  | Were there any concerns or fears that made you hesitant about the process? |  |
|  | How did your emotions influence your perioperative journey? | Did you feel scared or worried at any time? |
|  | Did the attitudes or behaviour staff impact your feelings about the surgery? What about friends or family? |  |
| Behaviour | What steps did you take to prepare for the surgery? | Did you have to manage your diet? Did you participate in an exercise program? Were any of these suggested to you? |
|  | Did the hospital provide any support or resources to prepare you for the surgery? | Physical or virtual contact with a doctor, allied health professional or nurse; provision of educational materials; information sessions; provision of an exercise program to improve fitness |
|  | Was a recovery care plan discussed with you before surgery? Were you able to follow this care plan? If not, what challenges did you face? |  |
|  | Did you seek help or support in other ways? | For example, talking to friends or family who had similar surgeries; viewing information online through reputable websites; connecting with others on social media e.g. facebook groups; independently seeking out advice from GP or Physiotherapist or other health professional; commenced an exercise program to improve fitness |
|  | What would be your preferred way of receiving support? Would it be in person, remotely or a combination of the two? |  |
|  | If you were asked to exercise while waiting for surgery, or given an exercise program, what is your preferred way of doing this? | Being supervised in-person while you exercise; receiving in-person or remote coaching by a health professional; being monitored remotely; be fully independent |
