# Supplementary material for: Development of a Cocreated Perioperative Joint Replacement Digital Care Pathway to Improve Surgical Outcomes Following Joint Replacement: Protocol for a Mixed Methods Study
Source: JMIR Res Protoc. 2025 Dec 4;14:e85701. doi: 10.2196/85701 (PMC12715470; doi:10.2196/85701)
Supplement: Multimedia Appendix 2 [file resprot_v14i1e85701_app2.docx]

## Interview Schedule Phase 3 Patients

## Demographics

| Age |  |
| --- | --- |
| Gender and Sex | ☐ Male ☐ Female ☐ Other |
| Surgery type |  |
| Aboriginal or TSI | ☐ Yes ☐ No ☐ Prefer not to say |
| Is English your primary language spoken at home? | ☐ Yes ☐ No |
| If no, what is your primary language spoken at home? |  |
| Employment Status | ☐ Full time ☐ Part time ☐ Casual ☐ Unemployed ☐ Retired |
| Partnered/non-partnered/Living arrangement |  |
| What is your highest education level? | ☐ Year 12 or equivalent ☐ Diploma ☐ Undergraduate ☐ Postgraduate ☐ Other |
| If other, what is your highest education level? |  |
| How long have you had your pain for? | ☐ Under one year ☐ 1 – <3 years ☐ 3 - <5 years ☐ 5 years or more |

| **Domain** | **Key Concept** | **Interview Questions** |
| --- | --- | --- |
| **Demand** (interest, intention, and use) | Evidence of expressed or actual use of the intervention | • How did you first hear about the digital care pathway, and what was your initial reaction? • Did you feel motivated to use the pathway during your perioperative journey? Why or why not? • In what ways did you use the pathway (e.g., information, reminders, exercise guidance, communication)? • How often did you use it, and what influenced whether you kept using it? • Would you want to continue using such a pathway for future surgeries or healthcare needs? |
| **Feasibility** (practicality of use and integration) | Extent to which the intervention can be carried out with intended users | • How easy or difficult was it for you to access and use the digital care pathway (e.g., login, navigation, technical requirements)? • Did you face any barriers (such as lack of time, internet access, device availability, or digital literacy)? • What support did you need (if any) to use the pathway effectively? • How well did the pathway fit into your daily routine and perioperative care? • Do you think most patients like you would be able to use it successfully? |
| **Acceptability** (satisfaction and willingness to use) | Perception of satisfaction, comfort, and perceived value | • How satisfied were you with the information and support provided through the pathway? • Did you feel comfortable and confident using it? • What aspects did you like the most? • What aspects did you find frustrating or unhelpful? • How does this digital pathway compare to other ways you have received perioperative care (e.g., face-to-face education, paper leaflets)? |
| **Appropriateness** (perceived fit and relevance) | Perceived suitability and compatibility with needs and setting | • Did the pathway content feel relevant to your surgery and recovery needs? • Did it provide the right amount of information (too much, too little, or just right)? • How well did it address your concerns, questions, or goals during the perioperative period? • Did you feel the digital pathway complemented or replaced other parts of your care? • In your opinion, does this type of pathway make sense as part of perioperative care? |
| **Closing Question** | Captures additional insights for improvement | • If you could change or improve one thing about the digital care pathway, what would it be? |
